# The association between dietary diabetic risk reduction score with anthropometric and body composition variables in overweight and obese women: a cross-sectional study

**DOI:** 10.1038/s41598-023-33375-w

**Published:** 2023-05-19

**Authors:** Mehdi Karimi, Farideh Shiraseb, Maryam Mofidi, Alireza Khadem, Sara Ebrahimi, Khadijeh Mirzaei

**Affiliations:** 1 Department of Clinical Nutrition, School of Nutritional Sciences and Dietetics, Tehran University of Medical Sciences (TUMS), Tehran, Iran; 2 Department of Community Nutrition, School of Nutritional Sciences and Dietetics, Tehran University of Medical Sciences (TUMS), P.O. Box: 14155-6117, Tehran, Iran; 3 Department of Nutrition, Science and Research Branch, Islamic Azad University, Tehran, Iran; 4 The Ritchie Centre, Hudson Institute of Medical Research, Monash University, Clayton, Melbourne, VIC Australia; 5 Food Microbiology Research Center, Tehran University of Medical Sciences, Tehran, Iran

**Keywords:** Endocrinology, Medical research, Endocrine system and metabolic diseases, Obesity, Nutrition

## Abstract

Dietary diabetes risk reduction score (DDRRs) is inversely associated with a lower risk of type 2 diabetes. Given the importance of the association between body fat and insulin resistance and the effect of diet on these parameters, this study aimed to investigate the association between DDRRS and body composition parameters, including the visceral adiposity index (VAI), lipid accumulation product (LAP), and skeletal muscle mass (SMM). This study was conducted on 291 overweight and obese women aged 18–48 years old recruited from 20 Tehran Health Centers in 2018. The anthropometric indices, biochemical parameters, and body composition were measured. A semi-quantitative food frequency questionnaire (FFQ) was used to calculate DDRRs. Linear regression analysis was used to examine the association between DDRRs and body composition indicators. The mean (SD) age of participants was 36.67 (9.10) years. After adjustment for potential confounders, VAI (β = 0.27, 95% CI = − 0.73, 1.27, P_trend_ = 0.052), LAP (β = 8.14, 95% CI = − 10.54, 26.82, P_trend_ = 0.069), TF (β = − 1.41, 95% CI = 11.45, 17.30, P_trend_ = 0.027), trunk fat percent (TF%) (β = − 21.55, 95% CI = − 44.51, 1.61, P_trend_ = 0.074), body fat mass (BFM) (β = − 3.26, 95% CI = − 6.08, − 0.44, P_trend_ = 0.026), visceral fat area (VFA) (β = − 45.75, 95% CI = − 86.10, − 5.41, P_trend_ = 0.026), waist-to-hip ratio (WHtR) (β = − 0.014, 95% CI = − 0.031, 0.004, P_trend_ = 0.066), visceral fat level (VFL) (β = − 0.38, 95% CI = − 5.89, 5.12, P_trend_ = 0.064), fat mass index (FMI) (β = − 1.15, 95% CI = − 2.28, − 0.02, P_trend_ = 0.048) decreased significantly over tertiles of DDRRs, and also there was no significant association between SMM and DDRRs tertiles (β = − 0.57, 95% CI = − 1.69, 0.53, P_trend_ = 0.322). The findings of this study demonstrated that participants with higher adherence to the DDRRs had lower VAI (β = 0.78 vs 0.27) and LAP (β = 20.73 vs 8.14). However, there was no significant association between DDRRs and VAI, LAP and SMM, which are mentioned as the primary outcomes. Future studies with larger sample of both genders are needed to investigate our findings.

## Introduction

Obesity which is increasing globally is a major risk factor for a wide range of chronic diseases^1^. The World Health Organization (WHO) has defined overweight and obesity as abnormal or excessive fat accumulation, a body mass index (BMI) ≥ 25 kg/m^2^ and ≥ 30 kg/m^2^, respectively^2^. According to the latest report by the WHO, over 1.9 billion adults were overweight, and of these, 650 million were obese in 2016^3^. Also, in 2016, the prevalence of overweight and obesity was 60.9% and 25.5% in Iran, respectively^4,5^. The results of several studies showed a higher prevalence of obesity in women. Furthermore, females with a higher BMI are at increased risk for breast cancer, atherosclerotic cardiovascular disease, hypertension, dyslipidemia, type 2 diabetes (T2D), and endocrine disorders^6–8^.

Obesity is commonly defined using BMI, while the evidence shows that this indicator is not a strong predictor of medical risks. Given the complicated function of adipose tissue, the distribution of lipids in different anatomic regions is more important for predicting diseases^9^. LAP and VAI, novel insulin resistance biomarkers are measured through anthropometric indices and metabolic parameters. LAP is calculated from waist circumference (WC) and fasting concentration of TGs, and VAI is calculated using the combination of BMI, WC, TGs, and high-density cholesterol (HDL)^10,11^. A systematic review and meta-analysis showed that, LAP is an inexpensive method to evaluate the risk of all-cause mortality, and hypertension. Also, it is an accurate indicator for diagnosing and evaluating diabetes, which can perform better than anthropometric indicators in this field^12^. Furthermore, another systematic review study reported a strong association between diabetes risk and LAP^13^.

The evidence has shown that lifestyle changes with diet modification are necessary to prevent obesity and its health outcomes^14,15^. Given foods and nutrients are consumed together, the dietary pattern approach enables researchers to examine the whole diet^16^. DDRRs was created by Rhee et al. to indicate a higher consumption of coffee, nuts, cereal fibre, and a high ratio of polyunsaturated fats (PUFA)/saturated fats (SFA), and a lower intake of high glycemic index (GI) foods, sugar-sweetened beverages (SSB), red and processed meats, and trans fatty acids^17^. While DDRRs includes lower GI foods and higher cereal fibre intake, which are components of a healthy diet and reduce the incidence of overweight and obesity, no previous study has examined the association between DDRRs with overweight and obesity in Iranian adults^18^.

Given the increasing prevalence of overweight and obesity and the importance of body composition as a key factor for predicting chronic conditions, this study for the first time assessed associations between DDRRs and LAP, VAI, and SMM in overweight and obese Iranian women.

## Materials and methods

### Study population

**Figure 1:**
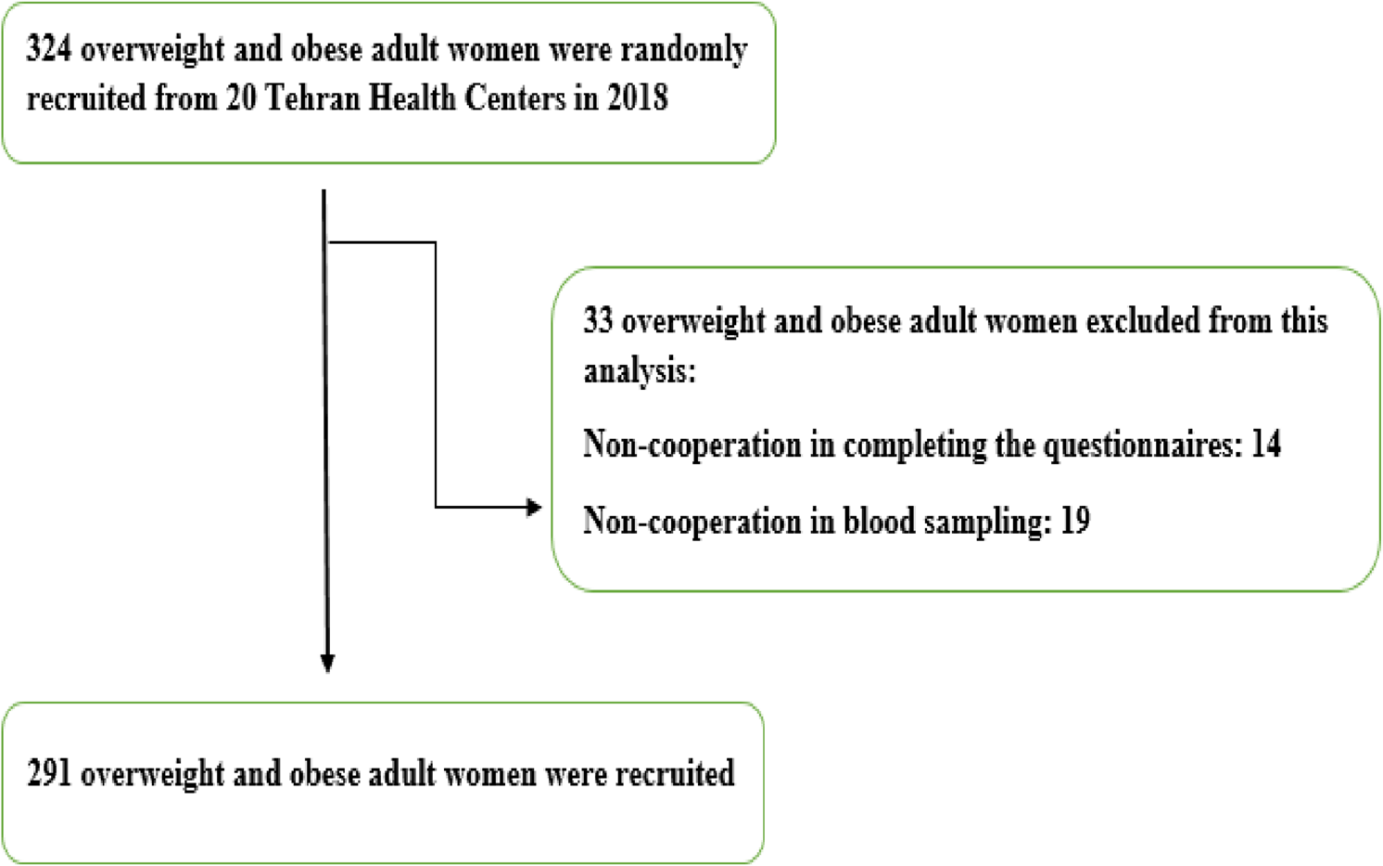
Flow chart of subjects’ enrolment.

This cross-sectional study used the multistage random sampling method and included 291 women aged 18–48 years old from 20 Tehran Health Centers in 2018. Indeed, 20 health centers were randomly selected from all health centers of the Tehran University of medical sciences (Fig. 1). The women who referred to Tehran health centers, if met the inclusion criteria, were randomly recruited to enter the study. The inclusion criteria were: consent to participate in the study, general health and not having a history of chronic disease mentioned in the exclusion criteria, and having BMI between ≥ 25 and ≤ 40. Exclusion criteria were regular use of oral contraceptives, medicines, and supplements including weight loss supplements or medication for blood lipids, blood sugar, and blood pressure reduction, diagnosed with diabetes mellitus, hypertension (HTN), impaired renal function, cardiovascular diseases (CVDs), and impaired liver function, smoking, alcohol consumption, pregnancy, lactation period, menopause, and the history of weight loss in recent years. Furthermore, participants who did not answer more than 70 questions of the semi-quantitative FFQ and reported daily energy intakes over 4200 kcal/day or lower than 800 kcal/day were excluded^19^. The protocol of this study was approved by the ethics committee of the Tehran University of Medical Sciences (IR.TUMS.VCR.REC.1395.1597). All methods were performed in accordance with the relevant guidelines and regulations, and all participants were fully informed about the study protocols and signed an informed consent form before participating. The sample size was computed according to the following formula: where *β* = 0.95 and *α* = 0.05, then, with 95% confidence and 95% power, and *r* = 0.37.

$${\text{n}} = [({\text{Z1 }}{-} \, \alpha + {\text{Z 1}} - \beta ) \times \surd {1} - {\text{r}}^{{2}} ]/{\text{r}})^{{2}} + {2})$$

### Sociodemographic characteristics

A demographic questionnaire was used to collect information on the medical history and current use of medications and supplement history, smoking habits, age, education, occupation, and marital status. The participant's level of physical activity was assessed using a validated international physical activity questionnaire (IPAQ)^20^. According to the IPAQ scoring criteria, physical activity was categorized into three levels: low (< 600 MET-min/week), moderate (≥ 600, < 3000MET-min/week), and high (≥ 3000 MET-min/week)^20^.

### Dietary intake assessment

A 147-item semi-quantitative FFQ was used to assess the usual dietary intake of participants. The validity and reliability of the FFQ have been previously demonstrated^21^. This questionnaire was completed by a trained dietitian. Participants reported the frequency of intake of a given serving of each food item over the last year on a daily, weekly, monthly, or yearly basis. Portion sizes of the food groups were converted to grams using household measurements, and individual’s dietary intake data were analyzed using the *Nutritionist* IV software^22^.

### Calculation of dietary diabetes risk reduction score

The DDRRs comprises eight components including a higher intake of cereal fibre, nuts, coffee, and PUFA to SFA ratio (P:S) and lower intake of red or processed meats, SSBs, trans fatty acids, and high GI foods^17^. To calculate the DDRRs, individuals were classified into quartiles according to their intake. For cereal fibre, nuts, coffee, and P:S ratio, the score range was between 1 and 4 assigned to the lowest and the highest intake, respectively. On the contrary, for red or processed meats, SSBs, trans fats, and high GI foods, scores between 1 and 4 were assigned to the highest and the lowest intake, respectively. The score of every component was summed up to calculate the total DDRRs score. The total DDRRs ranged between 8 (the lowest adherence) and 32 (the highest adherence)^23^. The DDRR score was categorized into tertiles. As a result, the score < 18 was the lowest, ≥ 18 to < 21 was the median, and ≥ 21 was the highest.

### Body composition

The body composition was measured using a multi-frequency BIA (InBody720, South Korea, the reliability of our BIA test–retest in our laboratory is r = 0.98) after 12 h of overnight fasting and according to the manufacturer's protocol precautions^24^. Participants were asked to remove extra clothes, including coat, sweater, shoes, and metal utensils/jewelry, such as rings, watches, and also avoid unusual physical activity for 72 h prior to the assessment. The body composition indicators including BMI, fat mass (FM), fat-free mass (FFM), BF%, visceral fat (VF), waist-to-hip ratio (WHR), bone mineral content (BMC), SMM, skeletal lean mass (SLM), FMI, lean trunk (LT), intracellular water (ICW) and extracellular water (ECW) were also measured^25^.

### Anthropometric indices

Anthropometric indices including weight, height, WC, and hip circumference (HC) were measured for each participant by a trained dietitian. Weight was measured using BIA, and height was measured with an accuracy of 0.1 cm using a Seca scale 206 while participants were in a standing position without shoes. WC was measured in the narrowest area of the waist and on bare skin without any pressure on the body, at the end of the natural exhalation, using a non-elastic tape with an accuracy of 0.5 cm. Using a strapless tape on the most prominent part that was marked, we measured the HC with an accuracy of 0.5 cm. To measure the arm circumference (AC), it was kept in a contracted position in line with the body and the elbow was bent 90° upwards, then its most prominent part was measured using a caliper. WHtR was calculated as WC (cm) divided by height (cm). All measurements were taken in  morning before breakfast and were performed by one person to reduce the measurement errors.

### LAP and VAI equations

VAI was calculated using sex-specific formulas, where both TGs and HDL levels are expressed in mmol/L^10^.

$$\begin{aligned} & {\text{Males}}: \, \left( {{\text{WC}}/{39}.{68} + \left[ {{1}.{88} \times {\text{BMI}}} \right]} \right) \times \left( {{\text{TGs}}/{1}.0{3}} \right) \times \left( {\left[ {{1}.{31}/{\text{HDL}}} \right]} \right). \\ & {\text{Females}}: \, \left( {{\text{WC}}/{36}.{58} + \left[ {{1}.{89} \times {\text{BMI}}} \right]} \right) \times \left( {{\text{TGs}}/0.{81}} \right) \times \left( {{1}.{52}/{\text{HDL}}} \right). \\ \end{aligned}$$

LAP was calculated as (WC/65) × TG in men, and (WC/58) × TG in women^26^.

### Blood sampling

Participants in this study were referred to the Nutrition and Biochemistry Laboratory of the School of Nutritional and Dietetics at Tehran University of medical sciences. After fasting for 10–12 h, 12 cm^3^ of venous blood samples were taken. Blood samples were collected in two tubes (one tube contained EDTA anticoagulant while another tube lacked this substance). The blood was centrifuged for 15 min at 3000 rpm, and the remaining blood was washed three times with 0.9% NaCl solution. Following serum separation, it was kept at − 80 °C for laboratory assessments.

### Blood pressure assessment and laboratory measurements

Before the blood pressure measurement, participants were asked about their intake of coffee and tea, as well as recent physical activity. Blood pressure was measured using a standard mercury sphygmomanometer, with appropriate cuffs, after 15 min of resting. A mean of two measurements was calculated for each individual^27^. The serum fasting glucose concentration was measured using an enzymatic colourimetric method with the glucose oxidase technique. The insulin level was assessed using the enzyme-linked immunosorbent assay (ELISA) kit (Human insulin ELISA kit, DRG Pharmaceuticals, GmbH, Germany). Serum TG level was measured using the glycerol-3-phosphate oxidase phenol 4-amino antipyrine peroxidase (GPO-PAP) method. ALT and AST were measured based on the standard protocols. Total cholesterol (CHOL) levels were assessed based on the enzymatic endpoint method. Low-density lipoprotein-cholesterol (LDL-C) and HDLC were measured using direct enzymatic clearance. All evaluations were performed using Pars Azmoon laboratory kits (Test Pars Inc, Tehran, Iran).

### HOMA and ISQUICKI calculations

Insulin resistance was measured using HOMA. The HOMA was calculated according to the following equation: HOMA = [Fasting Plasma Glucose (mmol/L) × Fasting Plasma Insulin (mIU/L)]/22.5^28^. Insulin sensitivity quantitative insulin sensitivity check index (ISQUICKI) was assessed based on the equation: ISQUICKI = 1/[log (fasting insulin) + log (fasting glucose)^29^.

### Statistical analysis

Statistical analysis was performed using the IBM SPSS software version 25.0 (SPSS, Chicago, IL, USA) and P-value < 0.05 was considered statistically significant and 0.05, 0.06, and 0.07 were considered marginally significant. Continuous and categorical variables were reported as means and standard deviations (SD), and number and percentage, respectively. The Kolmogorov–Smirnov test was used to determine the normal distribution of independent continuous variables (P > 0.05). A one-way analysis of variance (ANOVA) test was used to analyze continuous variables and a Chi-square test was used to compare qualitative variables according to tertiles of DDRRS. The analysis of covariance (ANCOVA) test was used to adjust the analysis for confounders and covariates including age, BMI, physical activity, and energy intake. Post-hoc (Bonferroni) analyses were performed to analyse the mean differences in continuous variables across tertiles of DDRRs. Linear regression analysis was used to examine associations between DDRRs and LAP, VAI, SMM, and other body composition components in the crude and adjusted models. The analysis was adjusted for potential confounders including age, energy intake, and physical activity in the first model and further for marital status and economic status in the second model. Findings were reported as Beta (β), standard error (SE), and 95% confidence intervals (CIs).

### Ethics approval and consent to participate and consent for publication

Ethics approval for the study protocol was confirmed by The Human Ethics Committee of Tehran University of Medical Sciences (Ethics Number: IR.TUMS.VCR.REC.1398.142). All participants signed a written informed consent that was approved by the Ethics committee.

## Results

### General characteristics of the study population

The characteristics of participants are presented in Table 1. The mean (SD) of age, BMI, FFM, VAI, and LAP of participants were 36.67 (9.10) years, 31.26 (4.29) kg/m^2^, 46.52 (5.71) kg, 2.46 (2.28) and 54.05 (41.72), respectively. The majority of participants were married (72.4%) and employed (99.5%).

**Table 1:** General characteristics according to tertiles of DDRRS in overweight and obese women (n = 291).

Variables	Tertiles of DDRRS			P-value*	P-value**
	T1	T2	T3		
	n = 101	n = 102	n = 88		
	< 18	18–21	> 21		
	Mean (SD)				
Age (year)	34.75 (8.94)	36.21 (8.60)	38.90 (7.35)^b^	**0.003**	**0.003**
PA (MET min/week)	726.85 (807.93)^a^	1715.88 (3213.59)	1154.88 (1304.81)	**0.008**	**0.006**
Body composition indicators					
Weight (kg)	82.24 (13.24)	80.89 (12.79)	78.74 (10.00)	0.142	0.858
Height (cm)	162.29 (5.78)	160.73 (5.81)	160.77 (6.14)	0.110	0.864
BMI (kg m^−2^)	31.22 (4.78)	31.40 (4.37)	30.46 (3.64)	0.293	0.823
WC (cm)	95.20 (17.79)	96.83 (16.17)	94.71 (14.12)	0.751	0.637
HC (cm)	114.59 (11.7)	114.71 (9.90)	13.20 (7.52)	0.660	0.862
AC (cm)	34.89 (4.00)	34.86 (3.41)	34.31 (2.77)	0.451	0.123
AMC (cm^2^)	28.26 (2.79)	28.69 (5.05)	27.95 (1.91)	0.358	0.168
TBW (kg)	34.84 (3.99)	34.30 (4.26)	33.91 (4.01)	0.290	0.645
ICW (kg)	21.53 (2.44)	21.17 (2.63)	20.93 (2.49)	0.257	0.598
ECW (kg)	13.36 (1.61)	13.13 (1.65)	13.01 (1.55)	0.314	0.713
BMC (kg)	2.70 (0.33)	2.64 (0.36)	2.64 (0.34)	0.373	0.940
Blood parameters					
FBS (mmol/L)	4.88 (0.53)	4.87 (0.52)	4.82 (0.55)	0.743	0.149
TG (mmol/L)	1.32 (0.56)	1.51 (0.96)	1.25 (0.75)^b^	**0.077**	**0.045**
HDL (mmol/L)	1.21 (0.24)	1.17 (0.29)	1.22 (0.28)	0.395	0.310
LDL (mmol/L)	2.39 (0.58)	2.42 (0.67)	2.48 (0.59)	0.652	0.953
TC (mmol/L)	4.78 (0.92)	4.72 (1.00)	4.84 (0.89)	0.729	0.349
AST (mg/dL)	17.10 (6.30)^a^	19.51 (8.52)^c^	16.80 (6.77)	**0.033**	**0.013**
ALT (mg/dL)	18.34 (12.13)	21.95 (15.13)^c^	17.09 (10.89)	**0.042**	**0.038**
Insulin (µIU/mL)	1.19 (0.24)	1.26 (0.24)^c^	1.18 (01.8))	**0.040**	**0.031**
HOMA_IR	3.42 (1.19)	3.44 (1.40)^c^	3.17 (1.24)	0.364	**0.063**
QIUKI (mg/L)	0.498 (0.025)	0.494 (0.022)	0.498 (0.025)	0.492	0.826
SBP (mm-Hg)	111.03 (13.6)	111.7 (12.79)	112 (15.03)	0.824	0.750
DBP (mm-Hg)	76.88 (9.50)	78.83 (8.63)	77.60 (10.73)	0.364	0.259

Categorical variables	N (%)				
Level of education				0.166***	0.923
Illiterate	2 (66.7)	1 (33.3)	0 (0.0)		
Under diploma	11 (30.6)	18 (50.0)	7 (19.4)		
Diploma	38 (35.5)	30 (28.0)	39 (36.4)		
Bachelor and higher	48 (33.8)	53 (37.3)	41 (28.9)		
Marriage status				0.091	**0.009**
Married	68 (32.4%)	71 (33.8%)	71 (33.8%)		
Single	31 (39.7%)	31 (39.7%)	16 (20.5%)		
Economic status				0.228	**0.061**
Poor	17 (25.4%)	24 (35.8%)	26 (38.8%)		
Moderate	50 (36.2%)	49 (35.5%)	39 (28.3%)		
Good	30 (41.7%)	25 (34.7%)	17 (23.6%)		
Occupational status				0.756	0.534
Employed	99 (34.7%)	100 (35.1%)	86 (30.2%)		
Unemployed	0 (0.0%)	1 (50.0%)	1 (50.0%)		
Supplement intake				0.959	0.563
Yes	47 (35.1%)	48 (35.8%)	39 (29.1%)		
No	36 (36.0%)	34 (34.0%)	30 (30.0%)		

*AC* arm circumference, *ALT* alanine transaminase, *AMC* arm muscle circumference, *AST* aspartate aminotransferase, *BMC* bone mineral content, *BMI* body mass index, *DBP* diastolic blood pressure, *DDRRs* dietary diabetes risk reduction score, *EBW* extracellular body water, *FBS* fasting blood sugar, *HDL-C* high-density lipoprotein cholesterol, *HOMA-IR* hemostatic model assessment for insulin resistance, *HC* hip-circumference, *IBW* intracellular body water, *QIUKI* quantitative insulin sensitivity check index, *LDL-C* low-density lipoprotein cholesterol, *SBP* systolic blood pressure, *SGOT* serum glutamic-oxaloacetic transaminase, *SGPT* serum glutamic-pyruvic transaminase, *PA* physical activity, *TBW* total body water, *TC* total cholesterol, *TG* triglyceride, *WC* waist circumference.

*P-value resulted from ANOVA analysis.

**P-value reported from ANCOVA, after adjustment for age, energy intake, physical-activity, and BMI. BMI was considered as colinear variable.

***P value resulted from Chi-square test analysis.

BMI was considered as collinear variable for anthropometric measurements and body composition.

P-value < 0.05 was considered significant, and 0.05, 0.06, and 0.07 were considered marginally significant.

^a^Significant difference was observed between T1 and T2.

^b^Significant difference was observed between T1 and T3.

^c^Significant difference was observed between T2 and T3.

Significant and marginally significant values are in bold.

### General characteristics across DDRRs tertiles

The general characteristics of participants over DDRRs tertiles are shown in Table 1. In the crude model, there was a significant mean difference in age (P = 0.003), physical activity (P = 0.008), TG (P = 0.077), AST (P = 0.033), ALT (P = 0.042), and insulin (P = 0.040) over DDRRs tertiles. After adjustment for potential confounders including age, energy intake, physical activity, and BMI, the mean difference remained significant for all variables (P < 0.05). Furthermore, HOMA-IR (P = 0.063), marriage status (P = 0.009), and economic status (P = 0.061) was significantly associated with DDRRs after controlling for confounding variables (age, energy intake, physical activity, and BMI).

### Dietary intake across tertiles of DDRRs

Table 2 represents the intake of nutrients and food groups across tertiles of DDRRs. There was no significant mean difference in macronutrients, including carbohydrate, protein, fat (P > 0.05) over DDRRs tertiles. A significant lower intake of SFA (P < 0.001) across tertiles of DDRRs was observed after adjustment for energy intake.

**Table 2:** Intake of macronutrients, micro-nutrients, and food groups according to tertiles of DDRRS in overweight and obese women (n = 291).

Variables	Tertiles of DDRRS			P-value*	P-value **
	T1	T2	T3		
	n = 101	n = 102	n = 88		
	< 18	18–21	> 21		
	Mean (SD)				
Energy (kcal/d)	2868.4 (703.8)	2579.0 (765.9)	2359.1 (700.6)	**< 0.001**	–
CHO (% energy)	56.13 (6.2)	56.45 (6.8)	56.93 (6.3)	0.705	0.700
Protein (% energy)	14.37 (2.9)	13.63 (2.3)	13.91 (2.5)	0.130	0.202
Fat (% energy)	32.47 (5.6)	32.55 (6.5)	32.26 (6.2)	0.950	0.922
Cholesterol (g/d)	281.96 (113.7)	248.03 (109.7)	223.48 (76.2)	**< 0.001**	0.172
SFA (g/d)	33.24 (11.5)	27.31 (10.9)	22.75 (7.6)	**< 0.001**	**< 0.001**
MUFA (g/d)	34.60 (10.1)	31.52 (14.1)	27.22 (9.2)	**< 0.001**	0.249
PUFA (g/d)	20.94 (8.7)	20.08 (10.3)	18.99 (7.8)	0.340	0.435
Linoleic acid (g/d)	18.07 (8.3)	17.40 (9.8)	16.40 (7.3)	0.415	0.482
Alpha-linolenic acid (g/d)	1.29 (0.65)	1.24 (0.73)	1.15 (0.63)	0.329	0.563
EPA (g/d)	0.031 (0.03)	0.030 (0.03)	0.035 (0.03)	0.569	0.417
DHA (g/d)	0.104 (0.115)	0.100 (0.114)	0.114 (0.115)	0.704	0.497
TFA (g/d)	0.008 (0.002)	0.001 (0.003)	0.0008 (0.001)	0.650	0.540
Sodium (mg/d)	4479.8 (1554.6)	4227.9 (1381.2)	3971. 9 (1274.3)	**0.049**	0.851
Potassium (mEq/d)	4426.3 (1567.6)	4168.2 (1437.0)	4334.5 (1660.1)	0.488	**< 0.001**
Vitamin A (mg/d)	797.09 (450.76)	734.39 (389.2)	780.22 (374.7)	0.528	0.067
Β-carotene	5090.3 (3952.4)	4865.5 (2923.3)	5758.6 (3569.4)	0.197	**0.005**
Vitamin C (mg/d)	210.54 (148.9)	187.89 (99.00)	184.95 (123.3)	0.296	0.451
Calcium (mg/d)	1245.98 (417.5)	1125.49 (386.9)	1096.52 (431.0)	**0.028**	0.375
Iron (mg/d)	19.52 (5.84)	18.36 (5.88)	17.79 (6.02)	0.123	**< 0.001**
Vitamin D (mg/d)	1.96 (1.82)	1.91 (1.44)	1.98 (1.55)	0.958	0.329
Vitamin E (mg)	17.97 (9.15)	17.45 (10.42)	16.34 (7.95)	0.478	0.836
Vitamin B_1_ (mg/d)	2.22 (0.62)	2.06 (0.65)	1.92 (0.65)	**0.006**	0.319
Vitamin B_2_ (mg/d)	2.40 (0.93)	2.11 (0.69)	2.02 (0.73)	**0.003**	0.546
Vitamin B_3_ (mg/d)	27.21 (10.15)	24.72 (9.04)	23.40 (7.52)	**0.013**	0.761
Vitamin B_6_ (mg/d)	2.28 (0.73)	2.10 (0.67)	2.06 (0.69)	**0.074**	**0.049**
Folate (mcg/d)	620.52 (164.15)	600.04 (173.84)	691.55 (190.06)	0.502	**< 0.001**
Vitamin B_12_ (mcg/d)	5.02 (2.77)	4.27 (2.38)	3.59 (1.59)	**< 0.001**	0.097
Biotin (mg/d)	38.95 (20.72)	36.53 (12.88)	39.07 (15.86)	0.488	**0.007**
Pantothenic acid (mg/d)	6.90 (2.85)	6.25 (1.85)	6.19 (2.26)	**0.068**	0.159
Vitamin K (mg/d)	204.85 (264.1)	212.33 (147.2)	217.5 (128.4)	0.901	0.507
Zinc (mg)	13.83 (4.23)	12.50 (3.89)	12.14 (4.30)	**0.012**	**0.079**
Phosphor (mg/d)	1741.01 (525.7)	1585.36 (476.1)	1551.99 (531.5)	**0.023**	**0.068**
Copper (mg/d)	2.05 (0.79)	1.95 (0.63)	1.94 (0.69)	0.523	**< 0.001**
Manganese (mg/d)	6.92 (2.43)	6.97 (2.75)	7.27 (3.26)	0.655	**< 0.001**
Magnesium (mg/d)	463.78 (143.5)	445.63 (140.2)	460.88 (159.7)	0.645	**< 0.001**
Selenium (mg/d)	126.45 (41.6)	119.07 (42.8)	112.21 (42.3)	**0.070**	0.295
Chromium (mg/d)	0.108 (0.08)	0.109 (0.08)	0.113 (0.08)	0.898	**0.066**
Total fiber (g/d)	44.73 (17.7)	45.26 (19.6)	45.31 (19.1)	0.972	**< 0.001**
Caffeine (g/d)	139.34 (100.4)	141.10 (103.6)	176.44 (226.7)	0.174	**0.027**
Food groups					
Whole grain (g/d)	5.54 (9.7)	6.81 (10.2)	10.8 (10.6)	**0.001**	**< 0.001**
Refined grain (g/d)	457.17 (180.1)	449.46 (247.6)	384.01 (222.7)	**0.046**	0.312
Fruits (g/d)	550.65 (333.4)	518.22 (306.04)	516.32 (379.22)	0.727	0.133
Vegetables (g/d)	407.55 (279.2)	430.34 (256.6)	467.18 (250.8)	0.297	**< 0.001**
Nuts (g/d)	12.11 (12.6)	13.24 (15.4)	18.26 (19.7)	**0.022**	**< 0.001**
Legumes (g/d)	44.32 (27.0)	50.61 (36.9)	64.70 (55.0)	**0.002**	**< 0.001**
Tea and coffee (g/d)	654.89 (490.4)	717.00 (534.2)	865.68 (1132.8)	0.151	**< 0.001**
SSB (g/d)	53.48 (94.3)	13.43 (28.3)	5.86 (22.2)	**< 0.001**	**< 0.001**
Dairy (g/d)	417.23 (245.8)	378.05 (259.8)	364.49 (229.7)	0.306	0.808
Eggs (g/d)	20.96 (15.7)	21.71 (14.05)	22.46 (12.3)	0.769	0.174
Fish and seafood (g/d)	11.15 (11.8)	11.67 (13.2)	11.39 (11.2)	0.954	0.508
Meat (g/d)	77.29 (56.4)	62.84 (52.5)	51.96 (34.23)	**0.002**	0.180

*EPA* eicosapentaenoic acid, *DHA* docosahexaenoic acid, *MUFA* monounsaturated fatty acid, *PUFA* polyunsaturated fatty acid, *SFA* saturated fatty acid, *SSB* sugar-sweetened beverages, *TFA* trans fatty acid.

*P-value resulted from ANOVA analysis.

**P-value reported from ANCOVA after adjustment for energy intake.

P-value < 0.05 was considered significant, and 0.05, 0.06, and 0.07 were considered marginally significant.

Significant and marginally significant values are in bold.

As shown in Table 2, after controlling for energy intake, there was a significant difference in the mean of potassium (P < 0.001), Β-carotene (P = 0.005), iron (P < 0.001), vitamin B_6_ (P = 0.049), folate (P < 0.001), biotin (P = 0.007), phosphor (P = 0.068), copper (P < 0.001), manganese (P < 0.001), chromium (P = 0.066), total fibre (P < 0.001), and caffeine (P = 0.027) over tertiles of DDRRs.

After adjustment for energy intake, participants with the highest tertile of DDRR score had a higher intake of whole grain, vegetables, nuts, legumes, tea and coffee (P < 0.001) and a lower intake of SSB (P < 0.001), compared to those in the lowest tertile.

### Anthropometric indices, VAI, and LAP across DDRRs tertiles

The association between anthropometric indices including BFM, FFM, VFA, SMM, VAI, and LAP over tertiles of DDRRs was presented in Table 3. While no significant mean difference in the crude model was observed, after controlling for confounders including age, energy intake, physical activity, marriage, and economic status, significant mean differences for VAI (P = 0.016) and LAP (P = 0.041) across tertiles of DDRRs were found. The results from Bonferroni posthoc test showed that the mean of VAI and LAP was higher in the first tertile compared to the second tertile.

**Table 3:** Primary outcomes including VAI, LAP, and muscle-mass across DDRRS tertiles in overweight and obese women (n = 291).

Variables	Tertiles of DDRRS			P-value
	T1	T2	T3	
	n = 101	n = 102	n = 88	
	< 18	18–21	> 21	
	Mean (SD)			
TF (kg)				
Crude	16.78 (1.84)	16.85 (3.99)	15.98 (3.12)	0.213*
Adjusted	16.28 (0.55)	16.34 (0.50)	15.88 (0.53)	0.751**
TF (%)				
Crude	313.3 (74.3)	320.02 (69.82)	306.98 (65.12)	0.443
Adjusted	302.90 (10.37)	306.98 (9.41)	301.71 (9.9)	0.921
BFM (kg)				
Crude	34.62 (9.55)	34.67 (9.05)	32.54 (6.91)	0.165
Adjusted	32.92 (1.13)	32.82 (1.03)	32.26 (1.08)	0.904
FFM (kg)				
Crude	47.41 (5.41)	46.66 (5.80)	46.19 (5.49)	0.315
Adjusted	46.10 (0.87)	47.09 (0.79)	46.86 (0.83)	0.695
SMM (kg)				
Crude	26.03 (3.16)	25.64 (3.44)	25.31 (3.28)	0.325
Adjusted	25.13 (0.53)	25.73 (0.49)	25.57 (0.50)	0.707
SLM (kg)				
Crude	44.69 (5.11)	44.00 (5.46)	43.20 (5.32)	0.158
Adjusted	43.46 (0.82)	44.38 (0.74)	44.14 (0.78)	0.705
BF (%)				
Crude	41.61 (5.50)	41.82 (5.98)	41.06 (5.05)	0.628
Adjusted	32.67 (1.26)	33.53 (1.17)	32.40 (1.19)	0.784
WHR (cm)				
Crude	0.93 (0.05)	1.83 (9.06)	0.92 (0.04)	0.395
Adjusted	0.937 (0.008)	0.935 (0.008)	0.927 (0.008)	0.679
VFA (cm^2^)				
Crude	181.62 (169.14)	163.85 (42.46)	158.05 (34.83)	0.266
Adjusted	160.91 (5.76)	158.67 (5.23)	156.56 (5.51)	0.870
VFL (cm)				
Crude	15.68 (3.35)	18.91 (23.45)	15.19 (3.22)	0.137
Adjusted	15.54 (0.53)	15.22 (0.48)	15.08 (0.50)	0.823
FFMI (kg/m^2^)				
Crude	17.99 (1.56)	19.30 (12.99)	17.83 (1.43)	0.354
Adjusted	17.58 (1.95)	20.70 (1.77)	18.14 (1.86)	0.441
FMI (kg/m)				
Crude	13.22 (3.65)	13.42 (3.41)	2.77 (3.04)	0.417
Adjusted	12.74 (0.47)	12.61 (0.42)	12.50 (0.44)	0.940
VAI				
Crude	1.97 (1.07)	2.94 (2.95)	2.41 (2.33)	0.103
Adjusted	3.82 (0.37)^a^	2.22 (0.33)	1.18 (0.35)	**0.016**
LAP				
Crude	45.73 (32.23)	59.98 (47.67)	55.86 (43.76)	0.213
Adjusted	49.88 (6.88)^a^	64.26 (6.25)	40.77 (6.58)	**0.041**

*BFM* body fat mass, *FFM* free fat mass, *FFMI* free fat mass index, *FMI* fat mass index, *LAP* lipid accumulation product, *SLM* soft lean mass, *SMM* skeletal muscle mass, *TF* trunk fat, *VAI* visceral adiposity index, *VFA* visceral fat areas, *VFL* visceral fat level, *WHR* waist hip ratio.

*P-value resulted from ANOVA analysis.

**P-value reported from ANCOVA after adjustment for age, energy intake, physical activity, marriage, and economic status.

P-value < 0.05 was considered significant, and 0.05, 0.06, and 0.07 were considered marginally significant.

^a^Significant difference was observed between T1 and T2.

^b^Significant difference was observed between T1 and T3.

^c^Significant difference was observed between T2 and T3.

Significant and marginally significant values are in bold.

### Associations between DDRRs and VAI, LAP, SMM, and anthropometric indices

The association between DDRRs and anthropometric indices is shown in Table 4. In the crude model, a significant positive association between DDRRs and VAI in tertile 2 (β: 0.96, 95% CI: 0.08, 1.83, P = 0.031) and a marginal inverse association between DDRRs and SLM in tertile 3 (β: − 1.49, 95% CI: − 2.99, 0.01, P = 0.053) was found. However, the significant association disappeared after adjustment for confounders (age, energy intake, physical activity, marital status, and economic status) in model 2. There was no significant association between DDRRs and LAP, trunk fat, BFM, FFM, SMM, BF%, WHR, VFA, VFL, FFMI, and FMI in the crude model (P > 0.05). However, after controlling for potential confounders in model 2, a negative association was found between DDRRs and trunk fat (P-value = 0.024), BFM (P-value = 0.023), BF% (P-value = 0.045), VFA (P-value = 0.0.26), and FMI (P-value = 0.045). There was no significant association between DDRRs and LAP, FFM, SMM, WHR, and VFL (P > 0.05). Furthermore, VAI (P_trend_ = 0.052) and LAP (P_trend_ = 0.069), TF (kg) (P_trend_ = 0.27), TF% (%) (P_trend_ = 0.074), BFM (P_trend_ = 0.026), WHR (P_trend_ = 0.066), VFA (P_trend_ = 0.026), VFL (P_trend_ = 0.064), FMI (P_trend_ = 0.048) decreased with increasing tertiles of DDRRs (Table 4).

**Table 4:** Association between DDRRS and VAI, LAP, SMM, and anthropometric variables in overweight and obese women (n = 291).

Variables	Tertiles	β	SE	95% CI	P-value	P-trend
LAP						
Crude	T2	14.25	8.10	− 1.75, 30.25	0.081	0.238
	T3	10.13	8.16	− 5.74, 26.00	0.211	
Model 1	T2	19.44	8.88	− 2.04, 36.85	0.129	**0.061**
	T3	11.99	9.15	− 5.95, 29.94	0.190	
Model 2	T2	20.73	9.03	3.01, 38.44	0.422	**0.069**
	T3	8.14	9.53	− 10.54, 26.82	0.393	
VAI						
Crude	T2	0.96	0.44	0.08, 1.83	**0.031**	0.375
	T3	0.43	0.44	− 0.43, 1.30	0.320	
Model 1	T2	0.99	0.50	− 0.30, 2.29	0.511	0.097
	T3	0.56	0.52	− 0.46, 1.58	0.282	
Model 2	T2	0.78	0.48	− 0.32, 2.24	0.124	**0.052**
	T3	0.27	0.51	− 0.73, 1.27	0.599	
TF (kg)						
Crude	T2	0.06	0.51	− 0.94, 1.08	0.895	0.150
	T3	− 0.79	0.53	− 1.84, 0.25	0.138	
Model 1	T2	0.03	0.55	− 1.06, 1.12	0.955	**0.064**
	T3	− 0.96	0.58	− 2.14, 0.15	**0.070**	
Model 2	T2	− 0.01	0.57	− 1.14, 1.12	0.986	**0.027**
	T3	− 1.41	0.62	11.45, 17.30	**0.024**	
TF (%)						
Crude	T2	6.66	9.80	− 2.55, 25.89	0.497	0.563
	T3	− 6.36	10.16	− 26.28, 13.55	0.531	
Model 1	T2	0.26	10.56	− 20.44, 20.97	0.980	0.160
	T3	− 15.76	11.11	− 37.54, 0.009	**0.056**	
Model 2	T2	0.20	10.95	− 21.26, 21.66	0.985	**0.074**
	T3	− 21.55	11.81	− 44.71, 1.61	**0.068**	
BFM (kg)						
Crude	T2	0.04	1.21	− 2.32, 2.42	0.969	0.107
	T3	− 2.07	1.25	− 4.53, 0.38	0.098	
Model 1	T2	− 0.07	1.28	− 2.60, 2.45	0.954	0.100
	T3	− 2.25	1.35	− 4.91, 0.40	**0.077**	
Model 2	T2	− 0.22	1.33	− 2.83, 2.39	0.867	**0.026**
	T3	− 3.26	1.43	− 6.08, − 0.44	**0.023**	
FFM (kg)						
Crude	T2	− 0.74	0.78	− 2.27, 0.78	0.338	0.129
	T3	− 1.22	0.80	− 2.80, 0.36	0.131	
Model 1	T2	0.10	0.87	− 1.61, 1.82	0.903	0.509
	T3	− 0.61	0.92	− 2.42, 1.19	0.504	
Model 2	T2	0.14	0.89	− 1.60, 1.89	0.871	0.296
	T3	− 1.02	0.96	− 2.91, 0.85	0.286	
SMM (kg)						
Crude	T2	− 0.39	0.46	− 1.29, 0.51	0.396	0.131
	T3	− 0.72	0.47	− 1.65, 0.21	0.133	
Model 1	T2	0.12	0.51	− 0.89, 1.13	0.816	0.489
	T3	− 0.38	0.54	− 1.45, 0.68	0.482	
Model 2	T2	0.16	0.52	− 0.86, 1.20	0.751	0.322
	T3	− 0.57	0.56	− 1.69, 0.53	0.309	
SLM (kg)						
Crude	T2	− 0.69	0.74	− 2.14, 0.76	0.350	**0.053**
	T3	− 1.49	0.76	− 2.99, 0.01	**0.053**	
Model 1	T2	0.06	0.83	− 1.57, 1.70	0.935	0.277
	T3	− 0.96	0.88	37.00, 45.03	0.271	
Model 2	T2	0.10	0.83	− 1.53, 1.74	0.899	0.274
	T3	− 1.01	0.90	− 2.78, 0.76	0.264	
BF (%)						
Crude	T2	0.21	0.77	− 1.31, 1.73	0.784	0.514
	T3	− 0.54	0.80	− 2.12, 1.02	0.495	
Model 1	T2	− 0.56	0.87	− 2.27, 1.15	0.522	0.196
	T3	− 1.19	0.92	− 2.99, 0.61	0.196	
Model 2	T2	− 0.59	0.90	− 2.36, 1.17	0.509	0.116
	T3	− 1.53	0.97	− 3.44, − 0.37	**0.045**	
WHR (cm)						
Crude	T2	0.89	0.74	− 0.57, 2.36	0.231	0.964
	T3	− 0.008	0.77	− 1.52, 1.51	0.991	
Model 1	T2	− 0.004	0.007	− 0.01, 0.01	0.634	0.199
	T3	− 0.011	0.008	− 0.02, 0.006	0.197	
Model 2	T2	− 0.003	0.008	− 0.019, 0.013	0.707	**0.066**
	T3	− 0.014	0.008	− 0.031, 0.004	0.122	
VFA (cm^2^)						
Crude	T2	− 17.76	14.65	− 46.49, 10.96	0.226	0.116
	T3	− 23.56	15.19	− 53.33, 6.21	0.121	
Model 1	T2	− 27.53	18.02	− 62.85, 7.79	0.127	**0.043**
	T3	− 38.32	18.95	− 75.48, − 1.16	**0.043**	
Model 2	T2	− 29.27	19.07	− 66.66, 8.11	0.125	**0.026**
	T3	− 45.75	20.58	− 86.10, − 5.41	**0.026**	
VFL (cm)						
Crude	T2	3.23	1.98	− 0.64, 7.11	0.103	0.873
	T3	− 0.48	2.05	− 4.51, 3.53	0.813	
Model 1	T2	4.03	2.47	− 0.82, 8.89	0.104	0.831
	T3	− 0.62	2.60	− 5.73, 4.48	0.811	
Model 2	T2	4.01	2.61	− 1.10, 9.13	0.124	**0.064**
	T3	− 0.38	2.81	− 5.89, 5.12	0.891	
FFMI (kg)						
Crude	T2	1.30	1.08	− 0.82, 3.44	0.230	0.941
	T3	− 0.15	1.13	− 2.37, 2.06	0.890	
Model 1	T2	1.42	1.36	− 1.24, 4.09	0.297	0.913
	T3	− 0.19	1.44	− 3.02, 2.63	0.893	
Model 2	T2	1.69	1.44	0.004, 4.51	**0.061**	0.995
	T3	− 0.05	1.56	− 3.12, 3.01	0.971	
FMI (kg)						
Crude	T2	0.20	0.47	− 0.73, 1.13	0.671	0.392
	T3	− 0.44	0.49	− 1.41, 0.52	0.367	
Model 1	T2	− 0.10	0.51	− 1.10, 0.90	0.845	0.136
	T3	− 0.81	0.54	− 1.87, 0.24	0.132	
Model 2	T2	− 0.16	0.52	− 1.99, 0.87	0.759	**0.048**
	T3	− 1.15	0.57	− 2.28, − 0.02	**0.045**	

*BFM* body fat mass, *CI* confidence interval, *FFM* free fat mass, *FFMI* free fat mass index, *FMI* fat mass index, *LAP* lipid accumulation product, *SE* standard error, *SLM* soft lean mass, *SMM* skeletal muscle mass, *TF* trunk fat, *VAI* visceral adiposity index, *VFA* visceral fat area, *VFL* visceral fat level, *WHR* waist hip ratio.

Tertile 1 of DDRRS was considered as a reference.

P-value reported using linear regression analysis.

Model 1 is adjusted for age, energy intake, and physical activity.

Model 2 is adjusted for model 1 + marital status and economic status.

Significant and marginally significant values are in bold.

## Discussion

According to our knowledge, this study is the first study investigated associations between DDRRs and LAP, VAL and SMM in overweight and obese women. According to our findings, there is an inverse and significant association between DDRRs and components of glycemic profiles (insulin, HOMA-IR), lipid profiles (TG), liver function enzymes (ALT, AST), and body composition indices (TF, BFM, FMI, BF%, VFA). Furthermore, body composition indices including VAI, LAP, TF, BFM, WHR, VFA, VFL, and FMI decreased significantly over DDRRs tertiles. However, no significant association was observed between VAI, LAP, and SMM and DDRRs.

The findings of this study showed a significant inverse association between DDRRs and BFM. In accordance with the results of our study, Perry et al. revealed that higher adherence to the DASH-style diet is associated with lower body fat in obese older American adults. The DASH diet was characterized by a higher intake of nuts, whole grains, fruits, vegetables, and legumes and a lower intake of carbonated beverages and red meat that is comparable to the components of DDRRs in this study^30^.

Our findings showed that the higher DDRRs is associated with a lower level of lipid profiles (serum triglycerides (TGs)), insulin profiles (insulin level and homeostasis model assessment-insulin resistance (HOMA_IR)), liver enzymes (aspartate aminotransferase (AST) and alanine transaminase (ALT)). In line with our findings, previous studies reported that higher adherence to the DASH diet was associated with improved lipid profiles, reduced TG and liver enzymes, and improved glycemic profiles, reduced serum insulin levels and HOMA-IR score^31^. The existing evidence showed that the Mediterranean diet characterized by a higher intake of healthy food groups including whole grains, MUFA, plant proteins, seafood, fruits, and vegetables, significantly reduced the BFM, which was consistent with the results of our study^32,33^. Furthermore, in agreement with the findings of this study, the evidence showed that the Mediterranean dietary pattern reduced weight, BMI, WC, fasting insulin levels, HOMA-IR, fatty liver indexes, TG, fasting plasma glucose, AST, and ALT^34,35^. In addition, in the direction confirming the results of our study, previous studies showed that participants in the lower tertiles compared to those in the higher tertiles of DDRRs, had higher HOMA-IR, triglycerides, and alanine transaminase as well as greater adiposity levels that could be due to higher intake of refined grains, sugary drinks, and saturated and trans-fat and lower intake of whole grains and PUFA^36,37^.

The higher intake of coffee, nuts, fibre, and PUFAs as components of DDRRs has been individually associated with lower BFM, lipid profiles, glycemic profiles, and liver enzymes. A recent study has reported that daily coffee consumption was inversely associated with BMI, BF%^38^, VFA^39^, total abdominal fat^39^, insulin and insulin resistance^40^, and levels of ALT and AST^41^. These associations could be explained through various mechanisms. Coffee comprises various components with pharmacologic effects, including caffeine and chlorogenic acid (CGA)^38^. Previous evidence revealed that CGA consumption increased postprandial energy expenditure and fat utilization in healthy participants and showed a suppressing effect on the accumulation of body fat^39,42,43^. There is also a possible explanation that antioxidants in coffee could improve insulin sensitivity and inhibit the induction of liver enzymes^40,44,45^. Furthermore, caffeine, an important chemical component of coffee, can reduce the risk of Type-2 diabetes and serum triglyceride levels^46,47^. However, the existing evidence regarding the effect of coffee is mixed. In a study conducted with a larger sample size of both genders in Greek adults, regular coffee consumption was negatively associated with VAL and LAP levels^48^. A systematic review suggests that adding nuts to habitual diets tends to lower body weight, FM and improve insulin sensitivity^46,47^. This effect might be explained by the fact that nuts comprise magnesium, linolenic acid, l-arginine, antioxidants, and MUFA may function against inflammation and insulin resistance^47^. Also, nuts are high-fibre, protein, and low-glycemic food groups, that cause weight loss through increasing satiety^49^. However, the evidence of the effect of nuts is inconsistent. A recent meta-analysis of randomized controlled trials demonstrated a diet with a higher intake of nuts had no significant impact on adiposity-related measurements compared to the control group^50^.

This study revealed that higher DDRRs is associated with a lower level of lipid profiles, insulin profiles and liver enzymes. A possible explanation may be that high fibre intake which is one of the components of DDRRs reduces body fat distribution^51^, lipid profiles^51,52^, fasting insulin, HOMA-IR score^53^, and liver function^54^. Furthermore, the low energy density of insoluble dietary fibre can improve postprandial satiety, lead to weight loss, and improves liver enzymes^55,56^. On the other hand, soluble fibre can reduce insulin resistance and inflammation^53^.

Opposite to our findings, which showed no significant association between DDRRs and VAL and LAP, Mazidi et al. reported that higher fibre intake in a healthy dietary pattern is associated with lower levels of VAL and LAP. The conflict results may be due to including a large number of participants from both genders in this study compared to our study, which included only women^57^.

As mentioned, this study showed that higher DDRRs is associated with lower lipid profiles, insulin profiles, and liver enzymes, which may be related to PUFAs as one of the components of DDRRs. Recent studies demonstrated that a high ratio of PUFA/SFA is associated with lower body fatness^58^, insulin resistance^59^, lipid metabolism^60^, and hepatic enzyme parameters^61^. Furthermore, it has been suggested that n-3 PUFAs may activate a metabolic change in adipocytes including increased β-oxidation, lipogenesis suppression in abdominal fat^62^, and inducing apoptosis in the adipose tissue (AT)^63^. Also, n-3 PUFAs activate the peroxisome proliferator-activated receptor (PPAR) alpha, which in turn stimulates fatty acid oxidation^64^, and PPAR gamma increases insulin sensitivity^65^, inhibits hepatic lipogenesis, and reduces hepatic reactive oxygen species^66^. While a randomized controlled trial study in 2021 showed that omega-3 (n-3 PUFAs) supplementation improved LAP and VAI levels, this study found no significant association which might be due to the fact that our study design was cross-sectional, while their study was a randomized controlled trial on diabetic patients with nonalcoholic fatty liver disease (NAFLD)^67^. Finally, it is likely that the anti-inflammatory, anti-atherogenic, decreasing visceral adiposity and improving dyslipidemia and hyperinsulinemia effects of DDRRs is due to its components, including antioxidants, vitamins and minerals, phenolic compounds, and unsaturated fatty acid^17,23^.

The current study has several limitations that should be considered in interpreting the results. Firstly, due to the cross-sectional design, causality cannot be conferred. As a result, further prospective observational studies and randomized clinical trials are needed to confirm the effect of DDRRs on LAP, VAL, and SMM. Secondly, using FFQs can result in under or over-reporting dietary intake. Thirdly, this study included only women; thus, it is impossible to generalize the results to the whole population. Lastly, using the categorical confounders might result in residual confounding. This study also has several strengths. This study is the first to show the link between DDRRs and LAP, VAL and SMM in adult women. This study included a large sample size and the analysis was controlled for various potential confounders.

## Conclusion

The findings of this study showed an inverse association between DDRRs and the percentage of BF, VFA, FMI, BFM, TF, serum TG and insulin level, HOMA_IR, AST, and ALT in overweight and obese women. While a higher adherence to DDRRs tertiles was negatively associated with lower VAL and LAP, DDRRs had no significant association with VAL, LAP, and SMM. Further prospective or interventional research is needed to confirm whether the association represents a cause-effect relationship.

## Data Availability

The data are not publicly available due to containing private information of participants. however, the data sets used and analyzed for the current study are available upon reasonable request of the corresponding author Dr. Khadijeh Mirzaei (mirzaei_kh@tums.ac.ir).

## Abbreviations

WHO: World Health Organization
BMI: Body mass index
T2D: Type 2 diabetes
LAP: Lipid accumulation product
VAI: Visceral adiposity index
WC: Waist circumference
TG: Triglyceride
HDL: High-density cholesterol
FFA: Free fatty acid
SMM: Skeletal muscle mass
DDRRs: Dietary diabetes risk reduction score
GI: Glycemic index
SSB: Sugar-sweetened beverages
CVD: Cardiovascular disease
FFQ: Food frequency questionnaire
BIA: Bioelectrical impedance analyzer
FM: Fat mass
FFM: Free-fat mass
BF%: Body fat percentage
VF: Visceral fat
BMC: Bone mineral content
SLM: Skeletal lean mass
FMI: Fat mass index
LT: Lean trunk
ICW: Intracellular water
ECW: Extracellular water
HC: Hip circumference
NC: Neck circumference
WHR: Waist-to-hip ratio
WHtR: Weight-to-height ratio
IPAQ: International Physical Activity Questionnaire
SBP: Systolic blood pressure
DBP: Diastolic blood pressure
CHOL: Total cholesterol
LDL-C: Low-density lipoprotein-cholesterol
HOMA: Homeostasis model assessment
ISQUICKI: Insulin sensitivity quantitative insulin sensitivity check index
MUFA: Monounsaturated fatty acid
FFMI: Free-fat mass index
BFM: Body fat mass
VFA: Visceral fat area
VFL: Visceral fat level
TF: Trunk fat
AST: Aspartate aminotransferase
ALT: Alanine transaminase
